# Transcriptional Response to a Dauer-Inducing Ascaroside Cocktail in Late L1 in *C. elegans*

**DOI:** 10.17912/micropub.biology.000397

**Published:** 2021-05-12

**Authors:** Sarah M Cohen, Jessica J Sun, Frank C Schroeder, Paul W Sternberg

**Affiliations:** 1 Division of Biology and Biological Engineering, California Institute of Technology, Pasadena, CA 91125, USA; 2 Boyce Thompson Institute, Ithaca, NY 14853, USA; 3 Department of Chemistry and Chemical Biology, Cornell University, Ithaca NY 14853, USA

## Abstract

The dauer diapause stage in *C. elegans* is a non-feeding alternative to the L3 larval stage that is highly resistant to harsh environmental conditions. The decision to enter dauer is a two-step process. First, L1 larvae encounter adverse conditions such as lack of food or overcrowding and decide to enter the L2d rather than the L2 stage. Second, L2d worms that continue to experience disadvantageous conditions decide to enter dauer instead of L3. Here, we have used RNA-seq to characterize the transcriptional response to a cocktail of dauer-inducing ascaroside pheromones at the late L1 stage as worms enter the L2d phase. We find that, in response to ascarosides, *C. elegans* L1 larvae preparing to enter the L2d stage begin upregulating genes involved in stress response and downregulating genes associated with growth and metabolism.

**Figure 1. Transcriptional response to ascarosides of C. elegans in early L1 stage:**
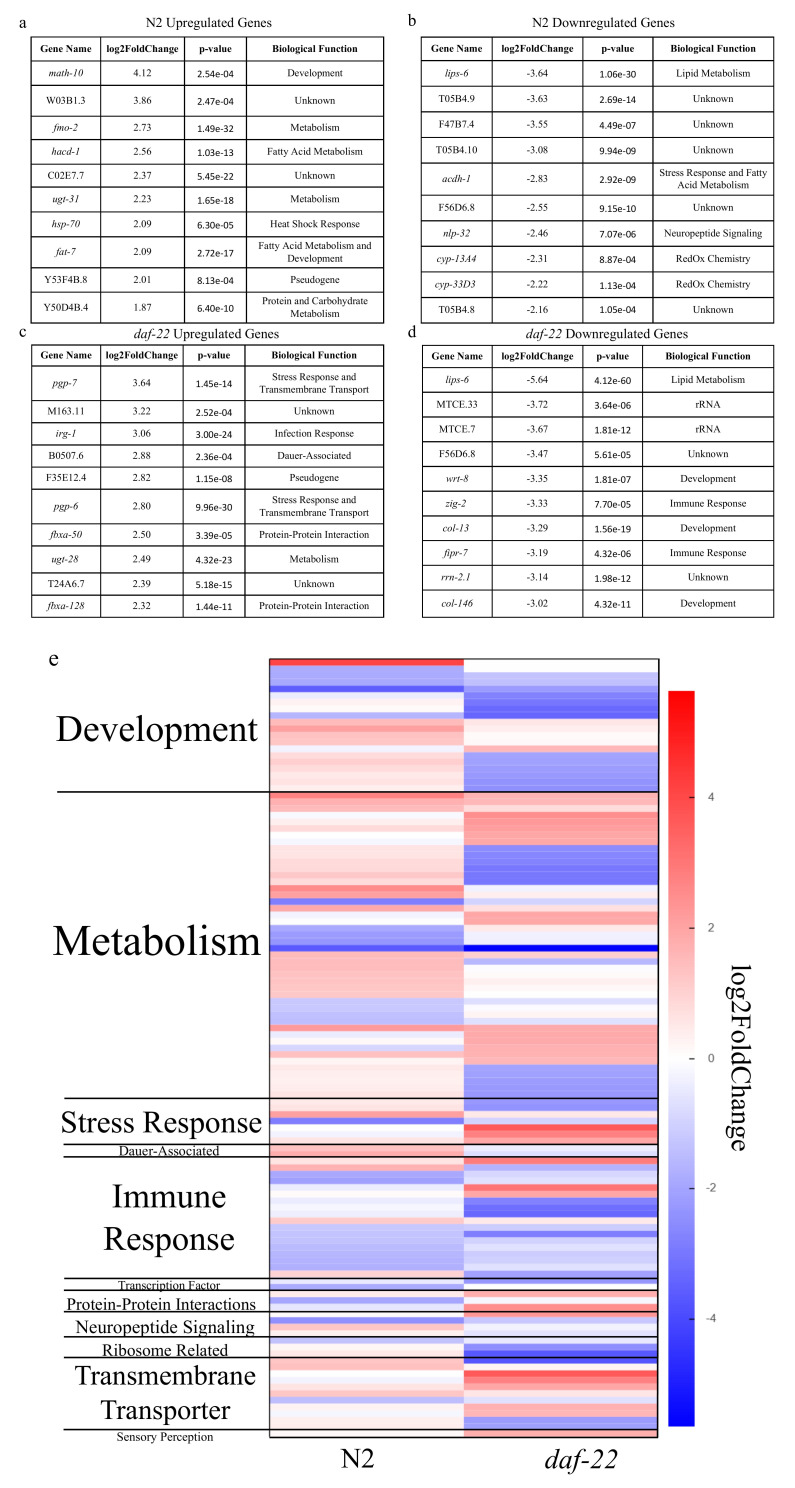
(a) The top ten up-regulated and (b) down-regulated genes in N2 (wild-type) L1 larvae. (c) The top ten up-regulated and (d) down-regulated genes in *daf-22* mutant L1 larvae. (e) A heatmap of the most significant genes at *p* = 0.001 with annotated biological functions, derived from WormBase Gene Ontology annotations, in the N2 and *daf-22* samples. A full list of the significant genes at *p* = 0.001 can be found in the Extended Data.

## Description

*C. elegans* larvae choose between dauer diapause and immediate reproductive development in two phases, the first being a choice between L2 and L2d, and the second between dauer and L3 (Golden and Riddle 1984). We used RNA-seq to study the transcriptomic response of well-fed *C. elegans* when exposed to a cocktail of diapause-inducing ascarosides (ascr#2, ascr#3, and ascr#8) during the L1 larval stage when they first receive cues from their environment and then adapt via developmental programming (Kaplan *et al.,* 2011). This research was performed in N2 wild-type worms and *daf-22(ok693)* mutants that lack an enzyme necessary for the biosynthesis of ascaroside pheromones (reviewed by Ludewig and Schroeder 2013). We found that early exposure to ascarosides triggers a pervasive transcriptional response that may drive diapause induction within as little as three hours. In N2, 362 genes were significantly differentially expressed at *p* = 0.001, while in *daf-22*, 1,007 genes were significantly differentially expressed at *p* = 0.001. The *daf-22* mutant may have more significantly expressed genes when exposed to ascarosides indicating a stronger response as it has never encountered ascaroside signaling previously, even at low levels.

A comparison of photographs of L1 larvae pre- and post-treatment with ascarosides did not show major differences in gonadal or intestinal development and size. However, analysis of the transcriptional data revealed significant changes in genes concerned with stress response (generally up-regulated) as well as growth and development (generally down-regulated), as one would expect as the worm prepares to enter L2d, a still-reversible precursor stage to the dauer diapause (Figure 1). This clearly shows that an ascaroside-induced transcriptional response occurs in the worms as they enter the L2 phase which begins to prepare them for the significant developmental changes of the dauer stage. In both N2 animals and *daf-22* loss-of-function mutants, the most highly down-regulated gene was *lips-6*, a putative lipase expressed in the intestine. *lips-6* has previously been found to be upregulated in starved adults and is activated via the same DAF-12/DIN-1 mechanism by which entry into the dauer diapause is regulated; in adults which no longer are capable of entering dauer, *lips-6* is activated instead (Tao *et al.,* 2016). That *lips-6* is downregulated so strongly in worms that prepare to enter dauer-diapause suggests that the starvation response pathways are temporal, and that *lips-6* is differentially used in the starvation response of L4 and adult worms as opposed to that of younger larval stages. The observation that expression of intestinal enzymes such as LIPS-6 is quickly downregulated in response to dauer-inducing ascarosides, which are perceived via sensory neurons, also indicates that neuronally perceived signals are quickly transduced to other tissues. Our results show that *C. elegans* responses to ascaroside pheromone signaling are analogous to the extensive cross-talk along the gut-brain axis in mammals.

## Methods

**Ascaroside Treatment**: Worms were grown in liquid cultures at 20ºC and 220 RPM using HB101 *E. coli* as a food source. Worms were synchronously grown in liquid cultures at a density of approximately one worm per uL of liquid and ~75mg/mL HB101 as described in Schaedel *et al.,* (2012). Briefly, for each genotype (N2 and the *daf-22* mutant), gravid adults were bleached to obtain a synchronous population of eggs. At six hours post-hatch, the original 60mL culture was separated into six equal 10mL cultures so that all experiments were done in triplicate. The three treatment cultures were each given 150uL of an ascaroside cocktail made of equal amounts (50uL) of 33uM synthetic ascr#2, ascr#3, and ascr#8 in ethanol, for a total ascaroside concentration of 0.5uM in each 10mL liquid culture. The three control cultures were given no ascarosides but were each given 150uL EtOH. The worms in liquid culture were exposed to the ascarosides or solvent for three hours before they were spun down, and the worm pellet was processed using a RNeasy kit to extract and purify the RNA in preparation for sequencing.

**Sequencing:** RNA integrity was assessed using RNA 6000 Pico Kit for Bioanalyzer and mRNA was isolated using NEBNext Poly(A) mRNA Magnetic Isolation Module. RNA-seq libraries were constructed using NEBNext Ultra RNA Library Prep Kit for Illumina following the manufacturer’s instructions. Briefly, mRNA isolated from ~1ug of total RNA was fragmented to the average size of 200nt by incubating at 94ºC for 15 minutes in first strand buffer, cDNA was synthesized using random primers and ProtoScript II Reverse Transcripatse followed by second strand synthesis using NEB Second Strand Synthesis Enzyme Mix. Resulting DNA fragments were end-repaired, dA tailed and ligated to NEBNext hairpin adaptors. After ligation, adaptors were converted to the ‘Y’ shape by treating with USER enzyme and DNA fragments were size selected using Agencourt AMPure XP beads to generate fragment sizes between 250 and 300 bp. Adaptor-ligated DNA was PCR amplified followed by AMPure XP bead clean-up. Libraries were quantified with Qubit dsDNA HS Kit and the size distribution was confirmed with High Sensitivity DNA Kit for Bioanalyzer. Libraries were sequenced on Illumina HiSeq2500 in single read mode with the read length of 50 nt and sequencing depth of 25 million reads per sample following manufacturer’s instructions.

**RNA-seq Data Analysis:** Data analysis was performed using the method described in Anders *et al.,* (2013). Briefly, sequence quality control was assessed using ShortRead and a metadata table of the raw fastq data files was created. The reference genome and gene model annotations were obtained from Ensembl (WBcel215), and a reference index was built using bowtie2. Reads were aligned to the reference genome using tophat2 and the BAM files were organized, sorted and indexed before SAM files were created using samtools. Reads were counted using HTSeq-Count and differential expression analysis was performed using edgeR and DESeq. The extended data for both N2 and *daf-22* includes the normalized counts for each replicate, the total list of differentially expressed genes, and the subset of significant genes at *p* = 0.001. Both our raw and processed data can be viewed at the Gene Expression Omnibus (GEO) – Accession Number: GSE171089.

## Reagents

Strains: The Bristol N2 strain was used as well as the *daf-22(ok693)* strain, obtained from the Caenorhabditis Genomics Center (CGC).

Ascarosides: ascr#2, ascr#3, and ascr#8 were synthesized in the Schroeder Lab (Pungaliya *et al.*. 2009).
