# Erratum: Transcriptional Response to a Dauer-Inducing Ascaroside Cocktail in Late L1 in C. elegans

**DOI:** 10.17912/micropub.biology.000466

**Published:** 2021-09-10

## Description

Cohen, SM; Sun, JJ; Schroeder, FC; Sternberg, PW (2021). Transcriptional Response to a Dauer-Inducing Ascaroside Cocktail in Late L1 in *C. elegans*. microPublication Biology. 10.17912/micropub.biology.000397. Erratum in: microPublication Biology. 10.17912/micropub.biology.000466.

The links to extended data files were omitted from the original article, this has been corrected. Links to the extended data files Cohen2021_N2_Ascr_extended and Cohen2021_daf-22_Ascr_extended have been attached to the original article.
